# Secreted Bacterial Effectors That Inhibit Host Protein Synthesis Are Critical for Induction of the Innate Immune Response to Virulent *Legionella pneumophila*

**DOI:** 10.1371/journal.ppat.1001289

**Published:** 2011-02-17

**Authors:** Mary F. Fontana, Simran Banga, Kevin C. Barry, Xihui Shen, Yunhao Tan, Zhao-Qing Luo, Russell E. Vance

**Affiliations:** 1 Department of Molecular and Cell Biology, Division of Immunology and Pathogenesis, University of California, Berkeley, Berkeley, California, United States of America; 2 Department of Biological Sciences, Purdue University, West Lafayette, Indiana, United States of America; Yale University School of Medicine, United States of America

## Abstract

The intracellular bacterial pathogen *Legionella pneumophila* causes an inflammatory pneumonia called Legionnaires' Disease. For virulence, *L. pneumophila* requires a Dot/Icm type IV secretion system that translocates bacterial effectors to the host cytosol. *L. pneumophila* lacking the Dot/Icm system is recognized by Toll-like receptors (TLRs), leading to a canonical NF-κB-dependent transcriptional response. In addition, *L. pneumophila* expressing a functional Dot/Icm system potently induces unique transcriptional targets, including proinflammatory genes such as *Il23a* and *Csf2*. Here we demonstrate that this Dot/Icm-dependent response, which we term the effector-triggered response (ETR), requires five translocated bacterial effectors that inhibit host protein synthesis. Upon infection of macrophages with virulent *L. pneumophila*, these five effectors caused a global decrease in host translation, thereby preventing synthesis of IκB, an inhibitor of the NF-κB transcription factor. Thus, macrophages infected with wildtype *L. pneumophila* exhibited prolonged activation of NF-κB, which was associated with transcription of ETR target genes such as *Il23a* and *Csf2*. *L. pneumophila* mutants lacking the five effectors still activated TLRs and NF-κB, but because the mutants permitted normal IκB synthesis, NF-κB activation was more transient and was not sufficient to fully induce the ETR. *L. pneumophila* mutants expressing enzymatically inactive effectors were also unable to fully induce the ETR, whereas multiple compounds or bacterial toxins that inhibit host protein synthesis via distinct mechanisms recapitulated the ETR when administered with TLR ligands. Previous studies have demonstrated that the host response to bacterial infection is induced primarily by specific microbial molecules that activate TLRs or cytosolic pattern recognition receptors. Our results add to this model by providing a striking illustration of how the host immune response to a virulent pathogen can also be shaped by pathogen-encoded activities, such as inhibition of host protein synthesis.

## Author Summary

In animals, the innate immune system senses infection primarily through detection of conserved microbial molecules. It has been suggested, but not clearly established, that the immune system may also respond to pathogen-associated activities—*i.e*., the manipulations of host cell processes that a pathogen employs to survive and replicate in its host. Previous studies have established that macrophages infected with the bacterial pathogen *Legionella pneumophila* can discriminate between virulent wildtype bacteria and an avirulent, nonreplicating mutant. Here we show that a unique host transcriptional response to virulent *L. pneumophila* is due to the activity of secreted bacterial proteins that inhibit host translation. Furthermore, we show that multiple bacterial toxins or chemicals that inhibit host translation can cooperate with host sensors of microbial molecules to induce the unique transcriptional response, even in the absence of bacterial infection. By demonstrating that the host mounts a response to a pathogen-encoded activity, we provide evidence for a novel mechanism of innate immune sensing that may aid in distinguishing pathogenic microbes from non-pathogens.

## Introduction

In metazoans, the innate immune system senses infection through the use of germline-encoded pattern recognition receptors (PRRs) that detect pathogen-associated molecular patterns (PAMPs), such as lipopolysaccharide or flagellin [1]. PAMPs are conserved molecules that are found on non-pathogenic and pathogenic microbes alike, and consequently, even commensal microbes are capable of activating PRRs [2]. Thus, it has been proposed that additional innate immune mechanisms may exist to discriminate between pathogens and non-pathogens [3], [4].

In plants, selective recognition of pathogens is accomplished by detection of the enzymatic activities of “effector” molecules that are delivered specifically by pathogens into host cells. Typically, the effector is an enzyme that disrupts host cell signaling pathways to the benefit of the pathogen. Host sensors monitoring or “guarding” the integrity of the signaling pathway are able to detect the pathogen-induced disruption and initiate a protective response. This mode of innate recognition is termed “effector-triggered immunity” [5] and represents a significant component of the plant innate immune response. It has been suggested that innate recognition of pathogen-encoded activities, which have been termed “patterns of pathogenesis” in metazoans [3], could act in concert with PRRs to distinguish pathogens from non-pathogens, leading to qualitatively distinct responses that are commensurate with the potential threat. However, few if any examples of “patterns of pathogenesis” have been shown to elicit innate responses in metazoans.

The gram negative bacterial pathogen *Legionella pneumophila* provides an excellent model to address whether metazoans respond to pathogen-encoded activities in addition to PAMPs. *L. pneumophila* replicates in the environment within amoebae [6], but can also replicate within alveolar macrophages in the mammalian lung [7], where it causes a severe inflammatory pneumonia called Legionnaires' Disease [6]. Because its evolution has occurred primarily or exclusively in amoebae, *L. pneumophila* appears not to have evolved significant immune-evasive mechanisms. Indeed, most healthy individuals mount a robust protective inflammatory response to *L. pneumophila*, resulting from engagement of multiple redundant innate immune pathways [8]. We hypothesize, therefore, that as a naïve pathogen, *L. pneumophila* may reveal novel innate immune responses that better adapted pathogens may evade or disable [9].

In host cells, *L. pneumophila* multiplies within a specialized replicative vacuole, the formation of which is orchestrated by bacterial effector proteins translocated into the host cytosol via the Dot/Icm type IV secretion system [10]. In addition to its essential roles in bacterial replication and virulence, the Dot/Icm system also translocates bacterial PAMPs, such as flagellin, nucleic acids, or fragments of peptidoglycan, that activate cytosolic immunosurveillance pathways [8], [11], [12], [13], [14], [15], [16]. There are also recent suggestions in the literature that Dot/Icm^+^ *L. pneumophila* may stimulate additional, uncharacterized immunosurveillance pathways [8], [17]. Overall, the molecular basis of the host response to Dot/Icm^+^ *L. pneumophila* remains poorly understood.

Here we show that macrophages infected with virulent *L. pneumophila* make a unique transcriptional response to a bacterial activity that disrupts a vital host process. We show that this robust transcriptional response requires the Dot/Icm system, and cannot be explained solely by known PAMP-sensing pathways. Instead, we provide evidence that the response requires the enzymatic activity of five secreted bacterial effectors that inhibit host protein synthesis. Effector-dependent inhibition of protein synthesis synergized with PRR signaling to elicit the full transcriptional response to *L. pneumophila*. The response to the bacterial effectors could be recapitulated through the use of pharmacological agents or toxins that inhibit host translation, administered in conjunction with a PRR agonist. Thus, our results provide a striking example of a host response that is shaped not only by PAMPs but also by a complementary “effector-triggered” mechanism that represents a novel mode of immune responsiveness in metazoans.

## Results

### Induction of an ‘effector-triggered’ transcriptional signature in macrophages infected with virulent *L. pneumophila*

**Figure 1:**
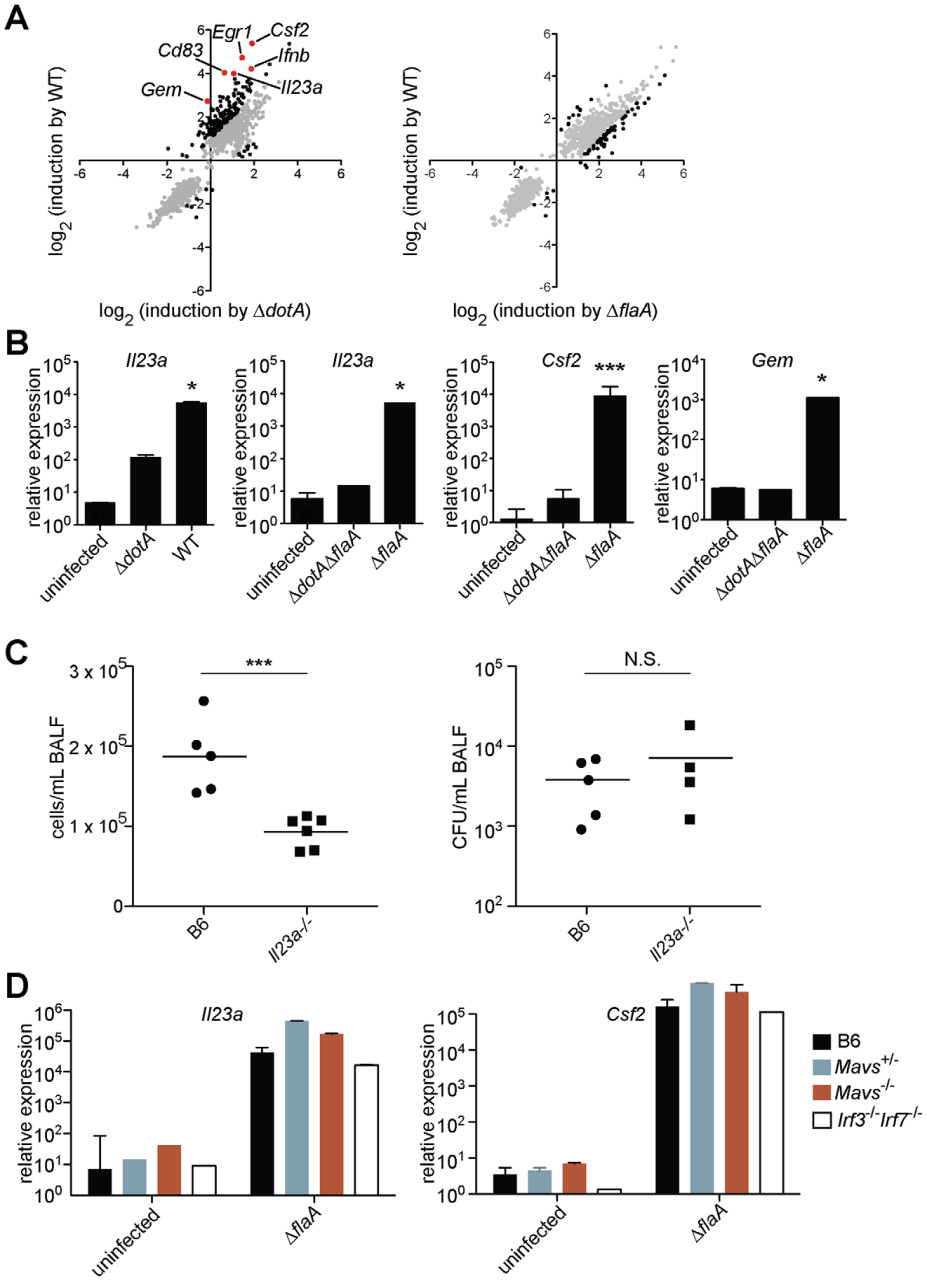
A unique transcriptional response in macrophages infected with virulent *L. pneumophila*. (**A**) *Caspase-1^−/−^* macrophages were infected for 6 h with the specified strains of *L. pneumophila*. RNA was amplified and hybridized to MEEBO microarrays. Black and red dots, genes exhibiting greater than 2-fold difference in induction between wildtype (WT) and mutant. Red dots indicate labeled genes. Data shown are the average of two experiments. (**B**) B6 macrophages were infected for 6 h with the specified strains of *L. pneumophila*. Levels of the indicated transcripts were measured by quantitative RT-PCR. (**C**) Mice were infected intranasally with 2×10^6^ *L. pneumophila* and bronchoalveolar lavage was performed 24 h post infection. Host cells recovered from bronchoalveolar lavage fluid (BALF) were counted with a hemocytometer. A portion of each sample was plated on BCYE plates to enumerate cfu. (**D**) Macrophages were infected for 6 h with *L. pneumophila*. Levels of the indicated transcripts were measured by quantitative RT-PCR. N.S., not significant. Data shown are representative of two (a, d) or at least three (B, C) experiments (mean ± sd in b, d). *, p<0.05 versus uninfected. ***, p<0.005 versus uninfected.

We initially sought to identify host responses that discriminate between pathogenic and non-pathogenic bacteria. Our strategy was to compare the host response to wildtype virulent *L. pneumophila* with the host response to an avirulent *L. pneumophila* mutant, Δ*dotA*. Δ*dotA* mutants lack a functional Dot/Icm secretion system, and thus fail to translocate effectors into the host cytosol, but they nevertheless express the normal complement of PAMPs that engage Toll-like receptor pathways. We performed transcriptional profiling experiments on macrophages infected with either wildtype *L. pneumophila* or the avirulent Δ*dotA* mutant. In the microarray experiments, *Caspase-1^−/−^* macrophages were used to eliminate flagellin-dependent macrophage death, which would otherwise differ between wildtype and Δ*dotA* infections [12], [14], [16], but our results were later validated with wildtype macrophages (see below). RNA was collected from macrophages at a timepoint when there were similar numbers of bacteria in both wildtype-infected and Δ*dotA*-infected macrophages. Microarray analysis revealed 166 genes that were differentially induced >2-fold in a manner dependent on type IV secretion (Figure 1A and Table S1). The induction of some of the Dot/Icm-dependent genes, *e.g*. *Ifnb*, could be explained by cytosolic sensing pathways that have been previously characterized [11], [13], [18]. However, much of the response to Dot/Icm^+^ bacteria did not appear to be accounted for by host pathways known to recognize *L. pneumophila*. For reasons discussed below, we refer specifically to this unexplained Dot/Icm-dependent transcriptional signature as the ‘effector-triggered response,’ or ETR.

The ETR includes many genes thought to be important for innate immune responses, including the cytokines/chemokines *Csf1*, *Csf2*, *Ccl20*, and *Il23a*; the surface markers *Sele*, *Cd83*, and *Cd44*; and the stress response genes *Gadd45*, *Egr1,* and *Egr3.* Other ETR targets were genes whose function in macrophages has not been determined (*e.g*., *Gem*, which encodes a small GTPase) (Figure 1A and Table S1). We selected several of the most highly induced genes for validation by quantitative reverse-transcription PCR. We confirmed that *Il23a*, *Csf2* and *Gem* transcripts were induced 100 to >1000-fold more by pathogenic wildtype *L. pneumophila* as compared to the Δ*dotA* mutant (Figure 1B). In subsequent experiments we focused on these three genes, as they provided a sensitive readout of the ETR.

To assess whether the ETR might be important during *L. pneumophila* infection *in vivo*, we infected B6 and *Il23a* ^−/−^ mice intranasally with *L. pneumophila*. *Il23a* ^−/−^ mice displayed a significant defect in host cell recruitment to the lungs 24 hours after infection (Figure 1C), consistent with the known role of IL-23 in neutrophil recruitment to sites of infection [19]. The phenotype of *Il23a* ^−/−^ mice was not due to decreased bacterial burden in these mice (Figure 1C). Thus at least one transcriptional target of the ETR plays a role in the host response, though there are clearly numerous redundant pathways that recognize *L. pneumophila in vivo* [8].

### Known innate immune pathways are not sufficient to induce the full ‘effector-triggered response’

**Figure 2:**
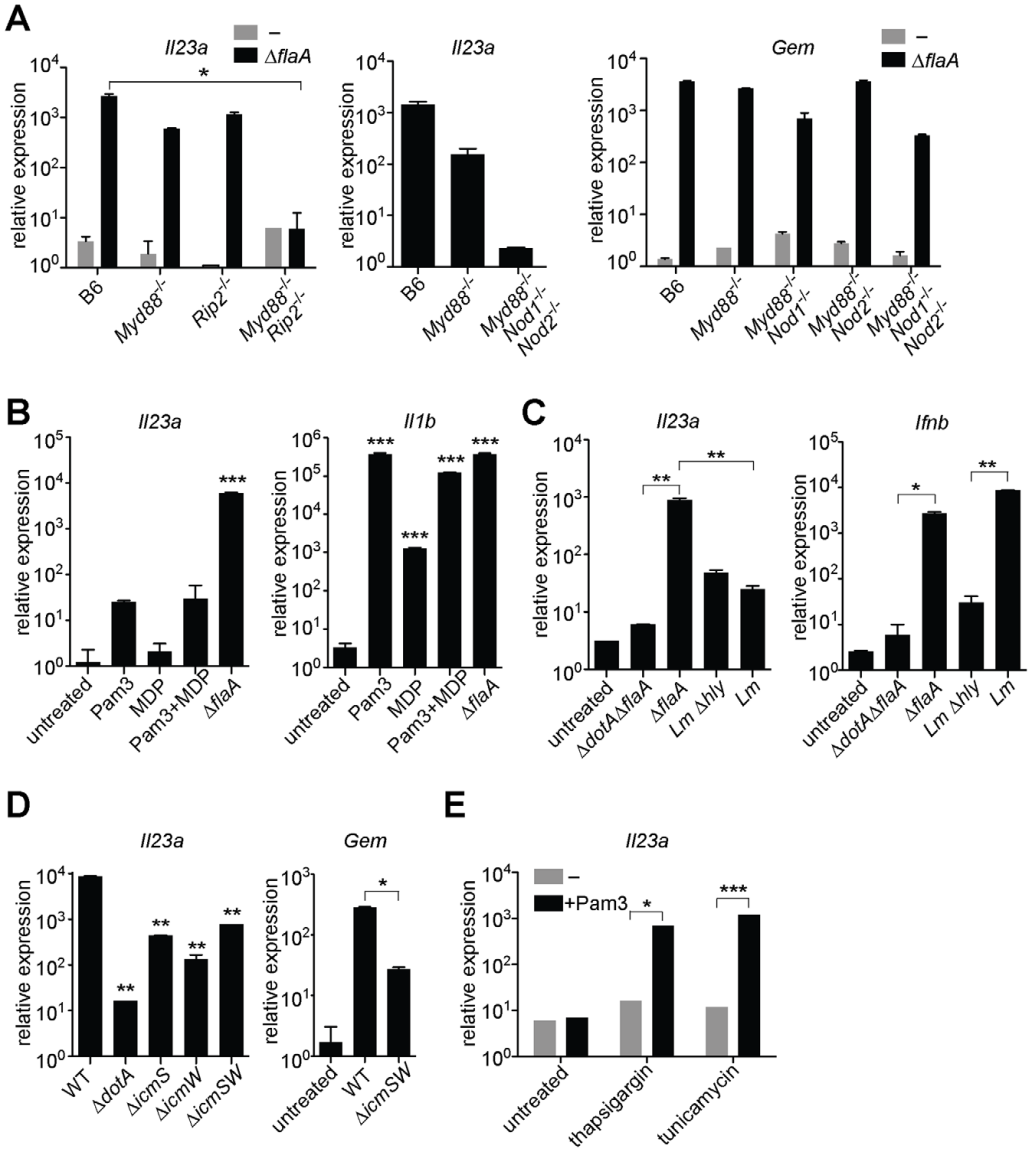
MyD88 and Nod signaling alone do not account for the unique response to virulent *L. pneumophila*, which can be recapitulated by ER stress inducers that also inhibit translation. In all panels, the indicated transcripts were measured by quantitative RT-PCR. (**A**) Macrophages were infected with Δ*flaA L. pneumophila* for 6 h. (**B**) Macrophages were infected with *L. pneumophila* or were treated with Pam3CSK4 (10 ng/mL) and/or transfected with MDP (10 µg/mL) for 6 h. (**C**) B6 macrophages were infected with *L. pneumophila,* wildtype *L. monocytogenes* or the avirulent *L. monocytogenes* Δ*hly* mutant for 4 h. (**D**) B6 macrophages were infected with the indicated strains of *L. pneumophila* for 6 h. **, p<0.01 compared to wildtype (WT). (**E**) Uninfected B6 macrophages were treated with thapsigargin (500 nM) or tunicamycin (5 µg/mL) for 6 h alone or in conjunction with Pam3CSK4 (1 ng/mL). All results shown are representative of at least three experiments (mean ± sd). Lm, *L. monocytogenes*. *, p<0.05; **, p<0.01; ***, p<0.005.

In order to identify the host pathway(s) responsible for induction of the ETR, we first examined innate immune pathways known to recognize *L. pneumophila*. Induction of the representative genes *Il23a*, *Csf2*, and *Gem* did not require the previously described Naip5/Nlrc4 flagellin-sensing pathway [20], as infection with a flagellin-deficient mutant (*ΔflaA*) also induced robust expression of these genes (Figure 1A, B and Table S2). Moreover, *Il23a, Csf2* and *Gem* were strongly (>1000-fold) induced in the absence of the Mavs/Irf3/Irf7 signaling axis shown previously to respond to *L. pneumophila* [11], [13], [18] (Figure 1D, and data not shown). As suggested by previous transcriptional profiling experiments [17], we confirmed that *Myd88* ^−/−^and *Rip2* ^−/−^macrophages, which are defective in TLR and Nod1/Nod2 signaling, respectively, strongly upregulated *Il23a* and *Gem* following infection with wildtype *L. pneumophila* (Figure 2A). Induction of *Il23a* was abrogated in *Myd88* ^−/−^ *Rip2* ^−/−^ and *Myd88* ^−/−^ *Nod1* ^−/−^ *Nod2* ^−/−^ macrophages; however, these macrophages still robustly induced *Gem* (Figure 2A, and data not shown). These data indicate that TLR/Nod signaling is necessary for induction of some, but not all, genes in the ETR. Furthermore, the intact induction of *Gem* in *Myd88* ^−/−^ *Nod1* ^−/−^ *Nod2* ^−/−^ macrophages implies the existence of an additional pathway.

To address the further question of whether TLR/Nod signaling was *sufficient* for induction of the ETR, we treated uninfected macrophages with synthetic TLR2 and/or Nod2 ligands (Pam3CSK4 and MDP, respectively). These ligands did induce low levels of *Il23a*, but could not recapitulate the robust (100–1000 fold) upregulation indicative of the ETR (Figure 2B). The defective induction of ETR target genes was not due to inefficient delivery of the ligands, as Pam3CSK4 and MDP were able to strongly induce *Il1b* (Figure 2B). To confirm this result in a more physiologically relevant system, we infected macrophages with the Gram-positive intracellular bacterial pathogen *Listeria monocytogenes*, which is known to activate both TLRs and Nods [21]. Infection with *L. monocytogenes* resulted only in weak *Il23a* induction (∼50 fold less than wildtype *L. pneumophila* at the same initial multiplicity of infection) (Figure 2C). A failure to strongly upregulate *Il23a* did not appear to be due to poor infectivity of *L. monocytogenes*, since the cytosolically-induced gene *Ifnb* [21] was robustly transcribed (Figure 2C). Taken together, these results suggest that TLR/Nod signaling, while necessary for transcription of some ETR targets, is not sufficient to account for the full induction of the ETR by *L. pneumophila*.

### Five *L. pneumophila* effectors that inhibit host protein translation are required to induce the full effector-triggered response

Though PRRs do play some role in induction of the ETR, we could not identify a known PAMP-sensing pathway that fully accounted for this robust transcriptional response. Therefore we considered the hypothesis that host cells respond to an *L. pneumophila*-encoded activity in addition to PAMPs. Since *L. pneumophila* manipulates host cell biology via its Dot/Icm-secreted effectors, we analyzed the transcriptional response of macrophages infected with *L. pneumophila ΔicmS/ΔicmW* mutants, which express a functional Dot/Icm system [15], but lack chaperones required for secretion of many effectors. Macrophages infected with *ΔicmS/ΔicmW L. pneumophila* exhibited a ∼50-fold defect in induction of *Il23a* and *Gem* (Figure 2D). Thus, secreted effectors (or the physiological stresses they impart) appear to participate in induction of the ETR.

To identify potential host pathways capable of inducing ETR target genes, we treated macrophages with known inducers of host cell stress responses. We found that the pharmacological agents thapsigargin and tunicamycin, which inhibit host translation via induction of endoplasmic-reticulum (ER) stress [22], synergized with a TLR2 ligand to induce high levels of *Il23a* and *Gem* (Figure 2E, and data not shown). To test whether *L. pneumophila* might elicit the ETR via induction of ER stress, we measured Xbp-1 splicing and transcription of classical ER stress markers in macrophages infected with *L. pneumophila.* However, we found no evidence of ER stress in these macrophages (data not shown). Instead, we considered the possibility that thapsigargin induces the ETR through inhibition of protein synthesis. In fact, the *L. pneumophila* Dot/Icm system was previously reported to translocate several effector enzymes that inhibit host translation [23], [24], [25]. Therefore we hypothesized that inhibition of host protein synthesis by *L. pneumophila* [26] might be responsible for induction of the ETR.

To determine whether inhibition of host translation by *L. pneumophila* was critical for induction of the ETR, we generated a mutant strain of *L. pneumophila*, called *Δ5*, which lacks five genes encoding effectors that inhibit host translation (*lgt1*, *lgt2*, *lgt3*, *sidI*, *sidL*; Figure S1; Table S3). Three of these effectors (*lgt1, lgt2, lgt3*), which share considerable sequence homology, are glucosyltransferases that modify the mammalian elongation factor eEF1A and block host translation both *in vitro* and in mammalian cells [23], [25]. A fourth effector (*sidI*) binds both eEF1A and another host elongation factor, eEF1Bγ, and has also been shown to inhibit translation *in vitro* and in cells infected with *L. pneumophila* [24]. The fifth effector, *sidL*, is toxic to mammalian cells and is capable of inhibiting protein translation *in vitro* via an unknown mechanism (data not shown). Moreover, its expression by *L. pneumophila* enhances global translation inhibition in infected macrophages (see below).

**Figure 3:**
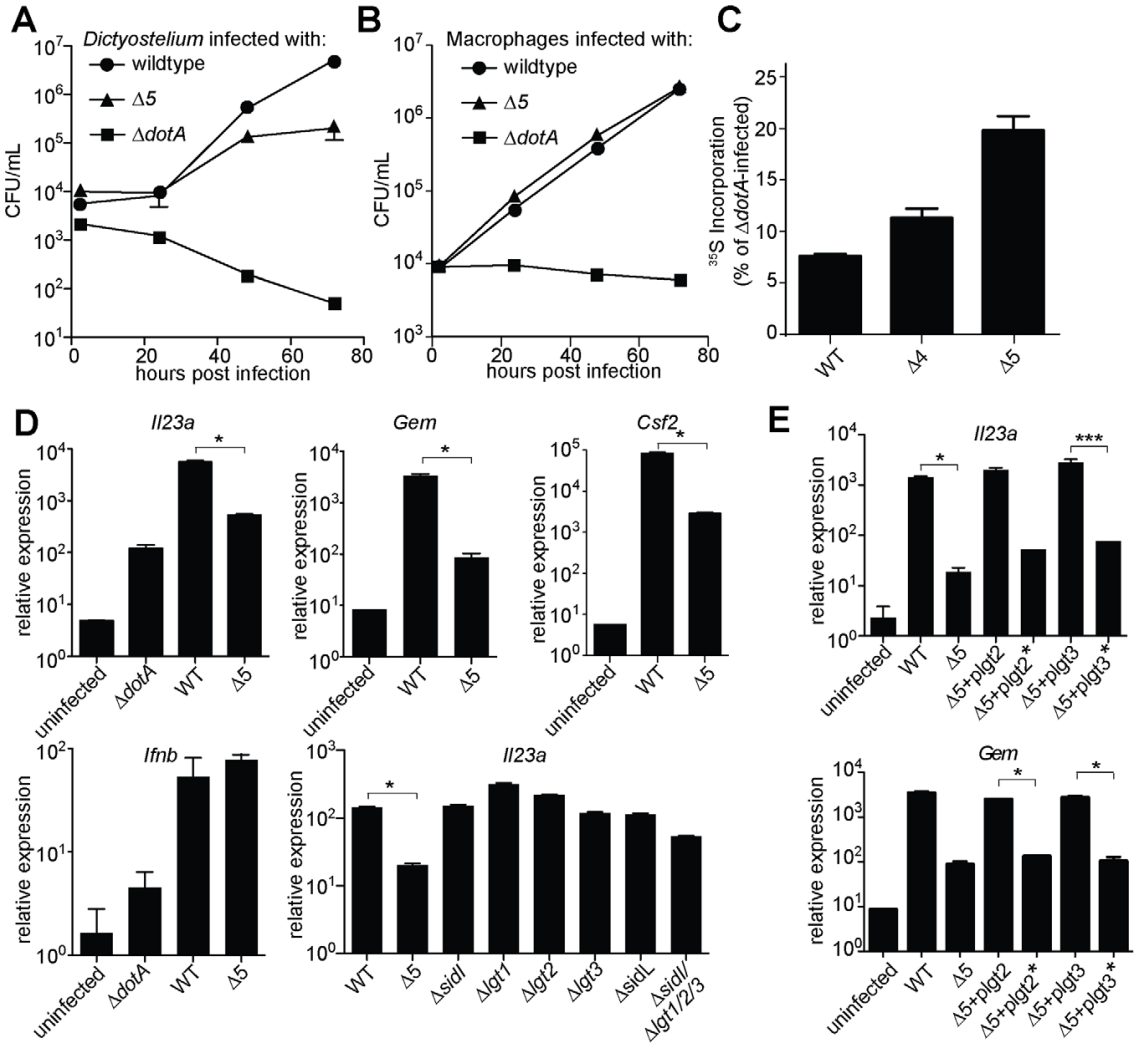
A mutant *L. pneumophila* lacking 5 bacterial effectors that inhibit host protein synthesis is defective in induction of the host ‘effector-triggered response’. Growth of the indicated strains of *L. pneumophila* was measured in amoebae (**A**) or A/J macrophages (**B**). (**C**) Global host protein synthesis was measured by ^35^S-methionine incorporation in macrophages infected for 2.5 h with the indicated strains. (**D**) *Myd88^−/−^* (bottom right graph) or *Caspase-1^−/−^* (all others) macrophages were infected for 6 h with the specified strains. The indicated transcripts were measured by quantitative RT-PCR. (**E**) *Caspase-1^−/−^* macrophages were infected for 6 h with the specified strains. Indicated strains carried plasmids that constitutively expressed either a functional (plgt2, plgt3) or a catalytically inactive (plgt2*, plgt3*) bacterial effector. Data shown are representative of two (b, c) or at least three (A, D, E) experiments (mean ± sd). Δ5, Δ*lgt1*Δ*lgt2*Δ*lgt3*Δ*sidI*Δ*sidL.* Δ4, Δ*lgt1*Δ*lgt2*Δ*lgt3*Δ*sidI*. *, p<0.05. ***, p<0.005.

These 5 effectors appear to be important for survival within the pathogen's natural host, since the *Δ5* mutant displayed a ∼10-fold growth defect in *Dictyostelium* amoebae (Figure 3A). By contrast, the *Δ5* mutant showed no growth defect in macrophages (Figure 3B), but was defective, compared to wildtype, in its ability to inhibit host protein synthesis (Figure 3C). Although to a lesser degree than wildtype bacteria, the *Δ5* mutant still appears to partially inhibit host protein synthesis, suggesting that *L. pneumophila* may encode additional inhibitors of host translation. Nevertheless, macrophages infected with *Δ5* exhibited striking defects in induction of the ETR, including a ∼50-fold defect in induction of *Il23a, Gem, and Csf2* (Figure 3D and Table S4). Importantly, the Dot/Icm-dependent induction of *Ifnb,* which is induced via a separate pathway [11], [13], [15], remained intact (Figure 3D), implying that the *Δ5* mutant was competent for infection and Dot/Icm function. Individual deletion mutants of each of the five effectors showed no defect in *Il23a*, *Csf2*, or *Gem* induction, whereas a mutant lacking four of the five (*Δlgt1Δlgt2Δlgt3ΔsidI*) had a partial defect (Figure 3D, and data not shown). Complementation of *Δ5* with wildtype *lgt2* or *lgt3* restored induction of *Il23a* and *Gem*, but complementation with mutant *lgt2* or *lgt3* lacking catalytic activity did not (Figure 3E). These results are significant because they show that macrophages make an innate response to a pathogen-encoded *activity* and that recognition of the effector molecules themselves is not likely to explain the ETR.

**Figure 4:**
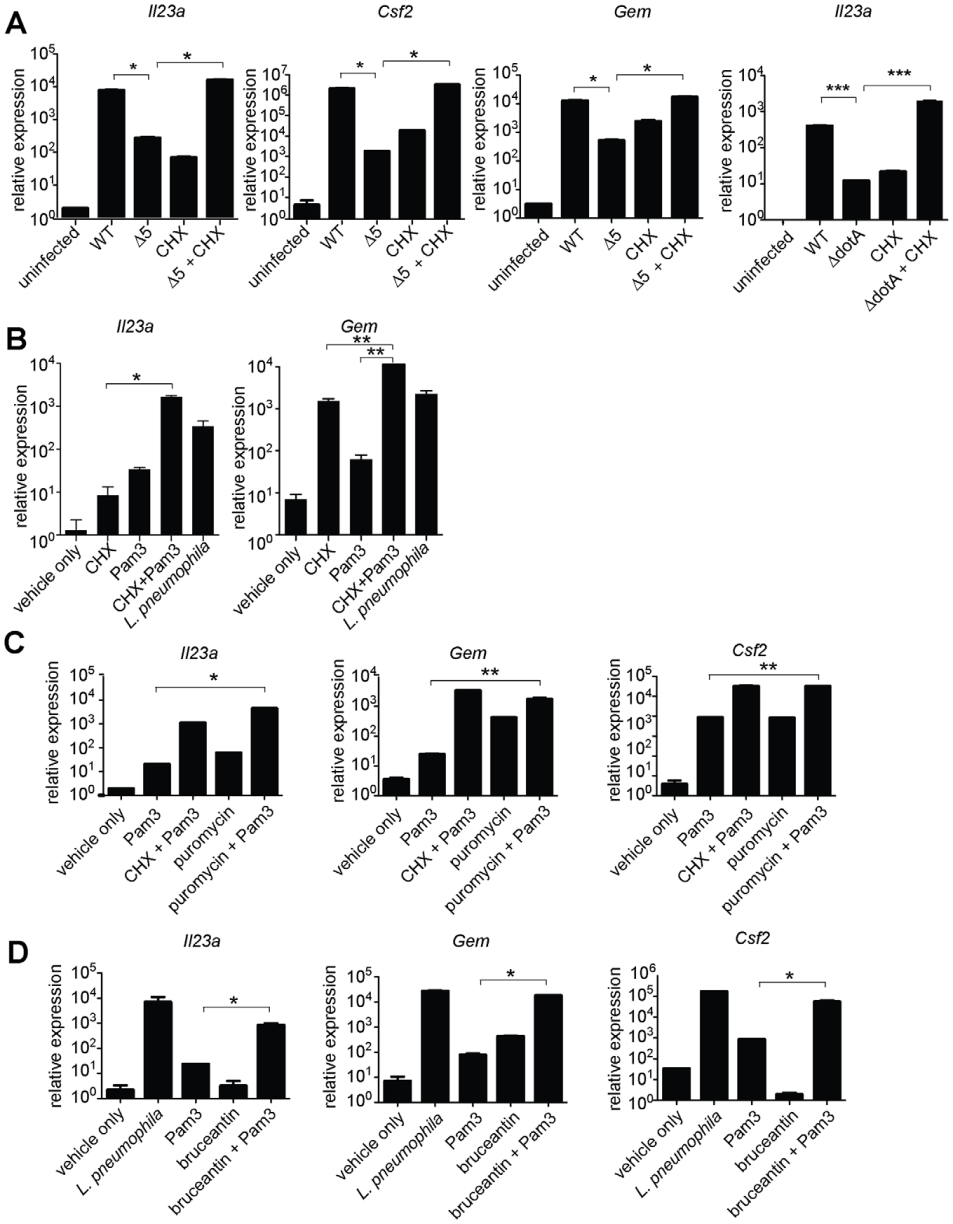
Induction of the ‘effector-triggered response’ can be recapitulated by pharmacological inhibitors of translation. (**A**) B6 macrophages were infected for 6 h with the indicated strains, alone or with CHX (5 µg/mL). (**B, C, D**) B6 macrophages were infected or were treated for 4 h with CHX (10 µg/mL; B), puromycin (20 µg/mL; C) or bruceantin (50 nM; D) alone or in conjunction with Pam3CSK4 (10 ng/mL). CHX, cycloheximide. Data shown are representative of two (C, D) or three (A, B) experiments (mean ± sd). *, p<0.05. **, p<0.01.

**Figure 5:**
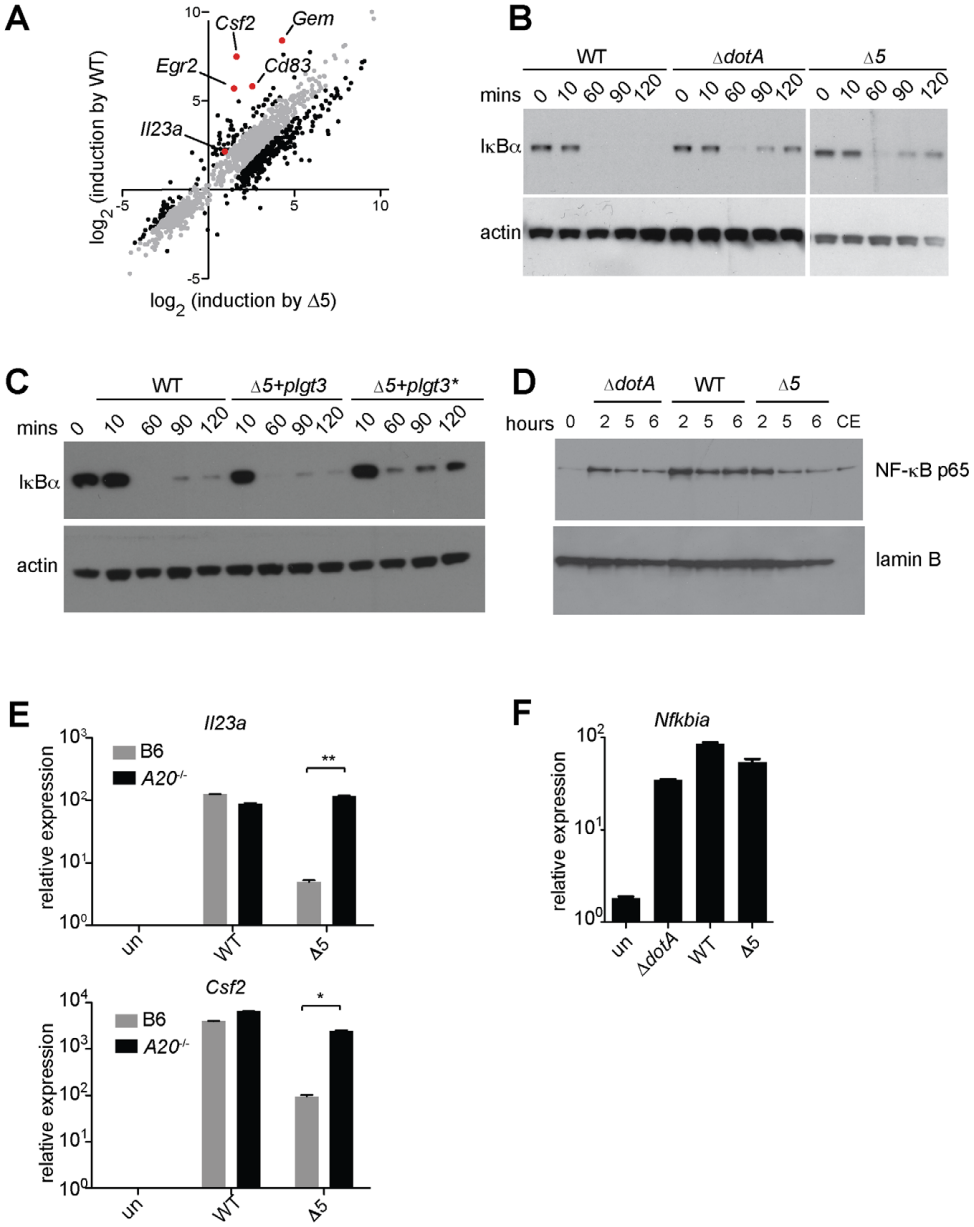
Expression of the 5 *L. pneumophila* effectors and induction of ‘effector-triggered’ genes correlates with sustained loss of inhibitors of the NF-κB transcription factor. (**A**) *Caspase-1^−/−^* macrophages were infected for 6 h with the indicated strains. RNA was amplified and hybridized to MEEBO arrays. Black and red dots, genes exhibiting greater than 2-fold difference in induction between wildtype (WT) and Δ*5*. Red dots indicate labeled genes. (**B, C**) *Caspase-1^−/−^* macrophages were infected at an MOI of 2 for the times indicated. Cell lysates were analyzed by Western blotting with anti-IκBα antibody (top panels) or anti-β-actin antibody (bottom panels). (**C**) The indicated strains carried a plasmid encoding either a functional (plgt3) or catalytically inactive (plgt3*) effector. (**D**) B6 macrophages were infected at an MOI of 2 for the times indicated. Nuclear extracts were analyzed by Western blotting with anti-NF-κB antibody (top panel) or anti-lamin-B antibody (bottom panel) as a loading control. Cytoplasmic extract of untreated macrophages (CE) was included for comparison. (**E, F**) B6 (E, F) or *A20^−/−^* (E) macrophages were infected for 6 h, and levels of the indicated transcripts were measured by quantitative RT-PCR. Data shown are representative of two experiments (E-F, mean ± sd).

We then tested more directly whether the ETR was induced by translation inhibition. The defective induction of *Il23a*, *Csf2,* and *Gem* in macrophages infected with *ΔdotA* or *Δ5* was rescued by addition of the translation inhibitor cycloheximide (Figure 4A, and data not shown). These results support the hypothesis that induction of the ETR by *L. pneumophila* involves inhibition of translation by the five deleted effectors. Importantly, the potent induction of *Il23a, Csf2* and *Gem* by *L. pneumophila* could be recapitulated in uninfected macrophages by treatment with the translation elongation inhibitors cycloheximide (Figure 4B) or puromycin (Figure 4C), or the initiation inhibitor bruceantin (Figure 4D), in conjunction with the TLR2 ligand Pam3CSK4. These three translation inhibitors possess different targets and modes of action, making it unlikely that the common host response to each of them is due to nonspecific drug effects. Thus, translation inhibition in the context of TLR signaling provokes a specific transcriptional response. Translation inhibitors alone were capable of inducing some, but not all, effector-triggered transcriptional targets (Figure 4B, C, and D), supporting our model that translation inhibition acts in concert with classical PRR signaling to generate the full effector-dependent signature. Microarray analysis indicated that the five effectors accounted for induction of at least 54 (∼30%) of the Dot/Icm-dependent genes (Figure 5A and Table S4).

### Inhibition of translation by *L. pneumophila* effectors results in sustained loss of IκB

We investigated how inhibition of protein synthesis by *L. pneumophila* might elicit a host response. Although translation inhibition by cycloheximide has long been reported to induce cytokine production [27], the mechanism by which it acts remains poorly understood. Since the induction of *Il23a* and *Csf2* is NF-κB dependent ([28], and data not shown), we examined a role for this pro-inflammatory transcription factor in induction of these ETR targets. NF-κB is normally suppressed by its labile inhibitor IκB, which is ubiquitinated and degraded in response to TLR and other inflammatory stimuli. IκB is itself a target of NF-κB-dependent transcription, and resynthesis of IκB is critical for the homeostatic termination of NF-κB signaling. In the absence of protein synthesis, we hypothesized that IκB may fail to be resynthesized as it turns over, thereby permitting continued NF-κB activity. To test this hypothesis, we measured IκB levels in infected macrophages over time. We observed a prolonged decrease in levels of IκB protein in macrophages infected with wildtype *L. pneumophila*, consistent with previous observations [17] (Figure 5B). In contrast, infection with Δ*5* triggered only a transient loss of IκB, similar to infection with the secretion-deficient Δ*dotA* mutant (Figure 5B). The Δ*5* mutant could induce sustained IκB degradation when complemented with plasmid-encoded *lgt3*, but not with a mutant effector lacking glucosyltransferase activity (Figure 5C), demonstrating that the sustained loss of IκB is due to the activity of the bacterial effector. To confirm that the prolonged loss of IκB did indeed result in sustained NF-κB activation, we measured NF-κB translocation to the nucleus in macrophages infected with wildtype, Δ*dotA,* or Δ*5 L. pneumophila*. While all three strains initially induced nuclear translocation of NF-κB, at later timepoints we observed decreased levels of nuclear NF-κB in macrophages infected with the Δ*dotA* or Δ*5* strains compared to those infected with wildtype *L. pneumophila* (Figure 5D). Thus, translation inhibition by the 5 effectors results in sustained loss of IκB and enhanced activation of NF-κB.

**Figure 6:**
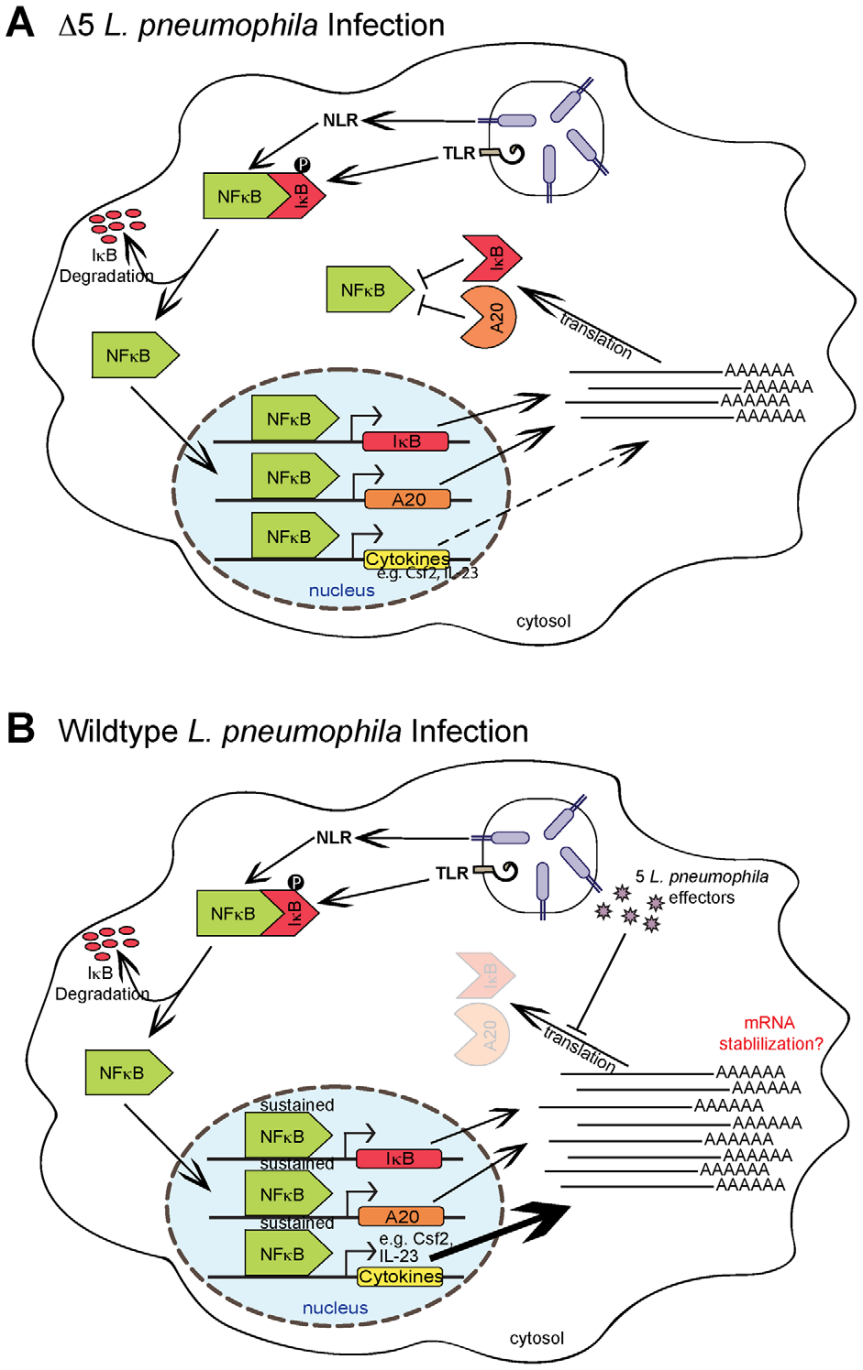
Model of NF-κB activation and superinduction by translation inhibitors. (**A**) NF-κB activation by TLR signaling, via the adaptor Myd88, or Nod signaling, via Rip2, normally leads to synthesis of inhibitory proteins, including IκB and A20, which act to shut off NF-κB signaling. (**B**) When translation is inhibited, IκB and A20 fail to be synthesized, allowing sustained activation of NF-κB and subsequent robust transcription of a subset of target genes.

NF-κB signaling is also inhibited by other *de novo* expressed proteins such as A20 [29]. We therefore used *A20* ^−/−^ macrophages, which exhibit prolonged NF-κB activation in response to TLR signaling [29], to further test the hypothesis that sustained NF-κB signaling can induce targets of the ETR. Strikingly, we found that the defective induction of *Il23a* and *Csf2* by Δ*5* was rescued in *A20* ^−/−^ macrophages (Figure 5E). Taken together, these observations suggest a model in which disrupted protein synthesis, and the subsequent failure to synthesize inhibitors of NF-κB signaling (*e.g.* IκB and A20), leads to sustained activation of NF-κB (Figure 6). In turn, we suggest that this prolonged activation of NF-κB results in enhanced transcription of a specific subset of genes.

Importantly, sustained NF-κB activation did not appear to result in transcriptional superinduction of all NF-κB-dependent target genes. Microarray analysis (Figure 5A and Table S4) suggested that only a subset of NF-κB-induced genes was preferentially induced by translation inhibition. For example, *Nfkbia* (encoding IκBα), a known NF-κB target gene, was not dramatically superinduced by wildtype compared to *Δ5 L. pneumophila* (Figure 5F). The molecular mechanism that results in specific superinduction of certain NF-κB-dependent target genes is not yet clear and may be complex (see Discussion). Inhibition of protein synthesis by *L. pneumophila* may also result in activation of other synergistic signaling pathways [30], such as MAP kinases ([17], data not shown), or in mRNA stabilization. In light of these possibilities, we confirmed that the increase in expression of ETR target genes does involve *de novo* transcription, by quantifying transcript levels using primers specific for unspliced mRNA (Figure S2A). We also tested whether mRNA stabilization contributed to induction of the ETR by infecting macrophages in the presence of the transcription inhibitor actinomycin D and quantifying ETR target mRNAs at successive timepoints. Our results suggested that RNA stabilization does not play a major role in induction of these particular ETR targets (Figure S2B), though we do not rule it out as a possible mechanism for increasing some mRNA transcripts in the ETR.

### Paradoxical increase in protein production under conditions where protein synthesis is inhibited

**Figure 7:**
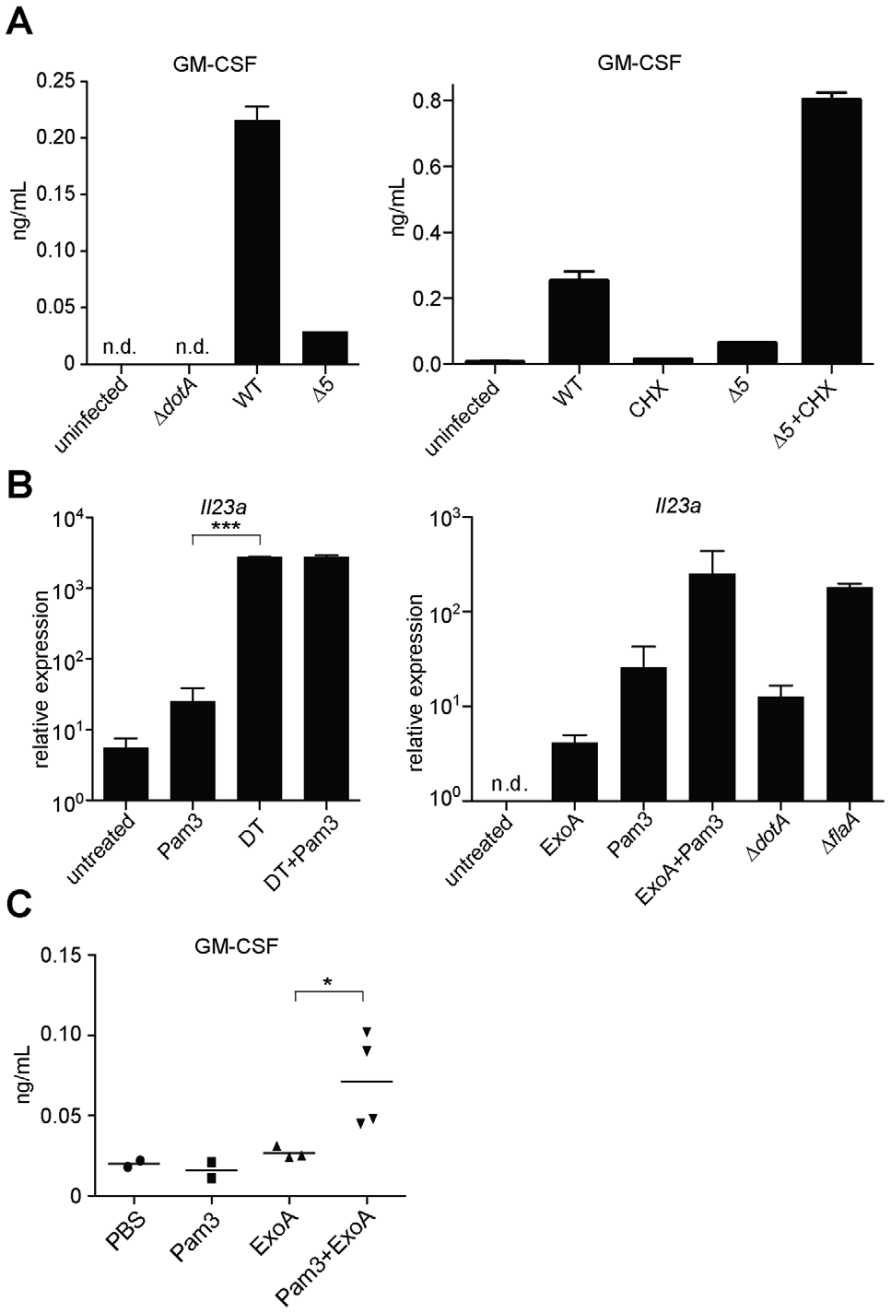
Inhibition of host translation by multiple bacterial toxins provokes an inflammatory cytokine response *in vitro* and *in vivo*. (**A**) B6 macrophages were infected for 24 h with the indicated strains of *L. pneumophila* and/or treated with cycloheximide (5 µg/mL). Protein levels in the supernatant were assayed by ELISA. (**B**) B6 macrophages were treated for 5 h with Diphtheria Toxin (1 ng/mL; left panel) or with Exotoxin A (500 ng/mL; right panel), alone or in conjunction with Pam3CSK4. *Il23a* transcript levels were assayed by quantitative RT-PCR. n.d., not detected. (**C**) B6 mice were treated intranasally with Pam3CSK4 (10 µg/mouse) or ExoA (2 µg/mouse) or both in 25 µL PBS. Bronchoalveolar lavage was performed 24 h post infection. GM-CSF levels in lavage were measured by ELISA. Data are representative of two (A, C) or three (B) experiments (mean ± sd in A, B). CHX, cycloheximide. DT, Diphtheria Toxin. ExoA, Exotoxin A. *, p<0.05. ***, p<0.005.

Although inhibition of protein synthesis potently induces transcription of certain target genes, a central question is whether this transcriptional response is sufficient to overcome the translational block, and result in increased protein production. Accordingly, we measured the protein levels of GM-CSF (encoded by the *Csf2* gene) in the supernatant of infected macrophages. GM-CSF protein was preferentially produced by cells infected with wildtype *L. pneumophila* as compared to cells infected with *Δ5* (Figure 7A). The defect in cytokine production by *Δ5-*infected macrophages was not due to poor bacterial growth (Figure 3B), increased cytotoxicity (Figure S3A), or defective secretion (Figure S3B), and could be rescued by addition of cycloheximide (Figure 7A). Thus translation inhibition can paradoxically lead to increased production of certain proteins, perhaps because transcriptional superinduction of specific transcripts is sufficient to overcome the partial translational block mediated by *L. pneumophila*.

### Host response to translation inhibition by bacterial toxins *in vitro* and *in vivo*

We did not observe defects in cytokine induction or altered bacterial replication in B6 mice infected with the *Δ5* mutant. This is perhaps not surprising, since many redundant innate immune signaling pathways are known to recognize and restrict the growth of *L. pneumophila in vivo* [8]. Indeed, we found that dendritic cells infected with *L. pneumophila* upregulate ETR target genes independently of the Dot/Icm secretion system (Figure S4), and hence translation inhibition appears not to be essential for their response to *L. pneumophila*.

However, many other pathogens also produce toxins that inhibit host protein synthesis (*e.g*., Diphtheria Toxin, Shiga Toxin, *Pseudomonas* Exotoxin A). Thus, to test whether translation inhibition may be a general stimulus that acts with PRRs to elicit a host response to diverse pathogens, we treated uninfected macrophages with Diphtheria Toxin (DT) or Exotoxin A (ExoA) in conjunction with a TLR2 ligand. Importantly, both of these toxins inhibit translation by ADP-ribosylation of eEF2, a mechanism of action distinct from that employed by the five *L. pneumophila* effectors. When administered with Pam3CSK4, both toxins robustly induced *Il23a* (Figure 7B). DT alone was sufficient to induce *Il23a*, most likely due to the presence of TLR ligands in the recombinant protein preparation. Consistent with these findings, Shiga Toxin, which inhibits translation by yet another mechanism, has also been reported to superinduce cytokine responses in a cultured cell line [31]. The existence of a common host response to diverse mechanisms of translation inhibition provides strong evidence that host cells can specifically respond to this disruption of their physiology, in addition to recognizing microbial molecules.

Finally, since *in vivo* infection with *L. pneumophila* results in multiple redundant responses that may have obscured our ability to detect an *in vivo* phenotype for the *Δ5* mutant, we turned to a simpler model to ascertain whether the ETR can be induced *in vivo*. In this model, purified Exotoxin A was administered intranasally to inhibit host protein synthesis in the lungs. Importantly, we found that translation inhibition appears to synergize with TLRs to elicit an immune response *in vivo*, as mice treated intranasally with ExoA and Pam3CSK4 produced significant amounts of the characteristic effector-triggered cytokine GM-CSF (Figure 7C). Consistent with our observations *in vitro* (Figure 7B), intranasal instillation of ExoA or Pam3CSK4 individually resulted in a much more modest response, providing further evidence that two signals—PRR activation and translation inhibition—are needed to generate the full effector-dependent signature. ExoA alone was sufficient to induce transcription of *Gem* and *Csf2* mRNA in the lung (Figure S5), again in agreement with *in vitro* observations that translation inhibition alone can induce transcription of some target genes (Figure 4B, C, and D). Taken together, our results demonstrate that translation inhibition by multiple pathogens can lead to a common innate response in cultured cells and *in vivo*.

## Discussion

In this study, we have demonstrated that inhibition of host translation by bacterial effectors or toxins can elicit a potent response from the host. We thus provide strong evidence for a model of innate immune recognition that is complementary to, but distinct from, the classic PAMP-based model. Most notably, we show that the immune system can mount a response to a pathogen-associated *activity*, in addition to pathogen-derived molecules. In our model, it is important to emphasize that there is no need for a specific host receptor or sensor *per se*. Instead, our data support the hypothesis that a pathogen-mediated block in the synthesis of short-lived host signaling inhibitors (*e.g*. IκB, A20) results in the sustained activation of an inflammatory mediator (*e.g.* NF-κB) (Figure 6). As such, our model more closely resembles the indirect “guard” type mechanisms that plants utilize, in conjunction with PRRs, to sense pathogens [5]. The labile nature of IκB makes it an effective “guard” to monitor the integrity of host translation, since the short half-life of this protein ensures that its abundance will decrease quickly during conditions where translation is inhibited.

There are growing suggestions that host responses to ‘patterns of pathogenesis’ [3], or harmful pathogen-associated activities, may indeed comprise a general innate immunosurveillance strategy in metazoans. For example, ion channel formation by influenza virus appears to activate the Nlrp3 inflammasome [32], and *Salmonella* effectors that stimulate Rho-family GTPases appear to trigger specific inflammatory responses [33]. However, in these examples, both the precise host cell disruption and the mechanism by which the host responds remain unclear. Our results are significant because we have provided a mechanism by which host cells generate a unique transcriptional response to a specific pathogen-encoded activity, namely, inhibition of host protein synthesis.

An important question is whether the innate response to translation inhibition represents a host strategy for detecting and containing a pathogen, or is rather a manipulation of the host immune system by the bacterium. Given the natural history of *L. pneumophila*, we consider it unlikely that this pathogen has evolved to manipulate the innate immune system [9]. *L. pneumophila* is not thought to be transmitted among mammals; instead, our data (Figure 3A) suggest that the five effectors described here probably evolved to aid survival in amoebae, the natural hosts of *L. pneumophila*. We therefore favor the hypothesis that the innate immune system has evolved to respond to disruptions in protein translation, an essential activity that is targeted by multiple viral and bacterial pathogens.

We observed that inhibition of translation in the context of PRR signaling results in the transcriptional superinduction of a specific subset of >50 genes, including *Il23a*, *Gem*, and *Csf2*, that constitute an ‘effector-triggered’ response. We propose that at least some of these genes are superinduced upon the sustained activation of transcription factors such as NF-κB, although it is important to emphasize that the host response to protein synthesis inhibition is complex and likely involves other pathways as well, such as MAP kinase activation (data not shown). Interestingly, we observed that not all NF-κB-dependent target genes are superinduced by translation inhibition. For example, *Nfkbia* (encoding the IκB protein) was not superinduced in wildtype *L. pneumophila* infection (Figure 5F). This selective superinduction of certain target genes may be significant, since it allows the host to respond to a pathogen-dependent stress by altering not only the magnitude but also the composition of the transcriptional response. Moreover, if IκB were superinduced, this would presumably act to reverse or prevent sustained NF-κB signaling, resulting in little net gain.

The mechanism by which prolonged NF-κB signaling may preferentially enhance transcription of the specific subset of effector-triggered genes is not yet clear. However, recent studies have shown that the chromatin context for several of these genes (*e.g*., *Il23a, Csf2*) is in a relatively ‘closed’ conformation [34], [35]. This may render the genes refractory to strong transcriptional induction under a normal TLR stimulus, but enable them to become highly induced upon prolonged NF-κB activation. It is interesting to note that genes such as *Il23a* and *Csf2* are classified as ‘primary’ response genes [34], [35] simply because they are inducible in the presence of cycloheximide. What is not often discussed is the possibility, demonstrated here, that inhibition of protein synthesis by cycloheximide is a key stimulus that induces transcription of these genes.

The consequences of the host response to translation inhibition are likely to be difficult to measure in the context of a microbial infection *in vivo*. Presumably, most pathogens that disrupt host translation derive benefit from this activity, perhaps by increasing availability of amino acid nutrients or by dampening production of the host response. These benefits may be offset by an enhanced host response to translation inhibition itself. It is possible that the robust innate immune response to translation inhibition serves primarily to compensate for the decrease in translation, resulting in little net change in the output of the immune response. Accordingly, the lack of an apparent phenotype during *in vivo* infection with *Δ5* may reflect the sum of multiple positive and negative effects that result from translation inhibition. Additionally, as suggested by our data (Figure S4) the response to *L. pneumophila in vivo* may involve non-macrophage cell types in which translation inhibition does not play a crucial role.

While PRR-based sensing of microbial molecules is certainly a fundamental mode of innate immune recognition, it is not clear how PRRs alone might be able to distinguish pathogens from non-pathogens, and thereby mount responses commensurate with the potential threat. Our results demonstrate that pathogen-mediated interference with a key host process (*i.e.*, host protein synthesis), in concert with PRR signaling, results in an immune response that is qualitatively distinct from the response to an avirulent microbe. Although induction of some genes in the ETR (*e.g*., *Gem*) occurs in response to inhibition of protein synthesis alone, much of the ETR is due to the combined effects of PAMP recognition and effector-dependent inhibition of protein synthesis. A requirement for two signals might be rationalized by the fact that the ETR includes potent inflammatory cytokines such as GM-CSF or IL-23, which can drive pathological inflammation [36] and autoimmunity [37] if expressed inappropriately. Restricting production of potentially dangerous cytokines to instances where a pathogenic microbe is present may be a strategy by which hosts avoid self-damage unless necessary for self-defense. Thus, we propose that the host response to a harmful pathogen-encoded activity may represent a general mechanism by which the immune systems of metazoans distinguish pathogens from non-pathogens.

## Materials and Methods

### Ethics statement

This study was carried out in strict accordance with the recommendations in the Guide for the Care and Use of Laboratory Animals of the National Institutes of Health. The protocol was approved by the Animal Care and Use Committee at the University of California, Berkeley (Protocol number R301-0311BCR).

### Mice and cell culture

Macrophages were derived from the bone marrow of the following mouse strains: C57BL/6J (Jackson Labs), *A20* ^−/−^ (A. Ma, UCSF), *Caspase-1* ^−/−^ (M. Starnbach, Harvard Medical School), *Mavs* ^−/−^ (Z. Chen, University of Texas SW), *Irf3/Irf7* ^−/−^ (K. Fitzgerald, U. Mass Medical School), *Myd88* ^−/−^ (G. Barton, UC Berkeley), *Rip2* ^−/−^ (M. Kelliher, U. Mass Medical School), *Myd88* ^−/−^ *Rip2* ^−/−^ (C. Roy, Yale University), and *Myd88* ^−/−^ *Nod1* ^−/−^ *Nod2* ^−/−^ (generated from crosses at UC Berkeley). *Il23a^−/−^* mice were from N. Ghilardi (Genentech). Macrophages were derived from bone marrow by 8d culture in RPMI supplemented with 10% serum, 100 µM streptomycin, 100 U/mL penicillin, 2 mM L-glutamine, and 10% supernatant from 3T3-M-CSF cells, with feeding on day 5. Dendritic cells were derived from B6 bone marrow by 6d culture in RPMI supplemented with 10% serum, 100 µM streptomycin, 100 U/mL penicillin, 2 mM glutamine, and recombinant GM-CSF (1:1000, PeproTech). *Dictyostelium discoideum* amoebae were cultured at 21°C in HL-5 medium (0.056 M glucose, 0.5% yeast extract, 0.5% proteose peptone, 0.5% thiotone, 2.5 mM Na_2_HPO_4_, 2.5 mM KH_2_PO_4_, pH 6.9).

### Bacterial strains

The *L. pneumophila* wildtype strain LP02 is a streptomycin-resistant thymidine auxotroph derived from *L. pneumophila* LP01. The *ΔdotA*, *ΔflaA*, *ΔicmS* and *ΔicmW* mutants have been described [14], [15]. Mutants lacking one or more effectors were generated from LP02 by sequential in-frame deletion using the suicide plasmid pSR47S as described [24]. Sequences of primers used for constructing deletion plasmids are listed in Table S3. Mutants were complemented with the indicated effectors expressed from the *L. pneumophila sidF* promoter in the plasmid pJB908, which encodes thymidine synthetase as a selectable marker. *L. monocytogenes* strain 10403S and the isogenic *Δhly* mutant have been described [21].

### Microarrays

Macrophage RNA from 1.5×10^6^ cells (6 well dishes) was isolated using the Ambion RNAqueous Kit (Applied Biosystems) and amplified with the Ambion Amino Allyl MessageAmp II aRNA Amplification Kit (Applied Biosystems) according to the manufacturer's protocol. Microarrays were performed as described [38]. Briefly, spotted microarrays utilizing the MEEBO 70-mer oligonucleotide set (Illumina) were printed at the UCSF Center for Advanced Technology. Microarray probes were generated by coupling amplified RNA to Cy dyes. After hybridization, arrays were washed, scanned on a GenePix 4000B Scanner (Molecular Devices), and gridded using SpotReader software (Niles Scientific). Analysis was performed using the GenePix Pro 6 and Acuity 4 software packages (Molecular Devices). Two independent experiments were performed. Microarray data have been deposited in the Gene Expression Omnibus database (http://www.ncbi.nlm.nih.gov/geo/) under the accession number GSE26491.

### Infection and stimulation

Macrophages were plated in 6 well dishes at a density of 1.5×10^6^ cells per well and infected at an MOI of 1 by centrifugation for 10 min at 400× *g*, or were treated with puromycin, thapsigargin, tunicamycin, cycloheximide (all Sigma), Exotoxin A (List Biological Labs), transfected synthetic muramyl-dipeptide (MDP) (CalBiochem), or a synthetic bacterial lipopeptide (Pam3CSK4) (Invivogen). Dendritic cells were plated at a density of 10^6^ cells per well and infected at an MOI of 2 as described above. Lipofectamine 2000 (Invitrogen) was used for transfections. Bruceantin was the kind gift of S. Starck and N. Shastri (UC Berkeley), who obtained it from the National Cancer Institute, NIH (Open Repository NSC165563). A fusion of diphtheria toxin to the lethal factor translocation signal (LFn-DT) was the gift of B. Krantz (UC Berkeley) and was delivered to cells via the pore formed by anthrax protective antigen (PA) as described [39].

### Quantitative RT-PCR

Macrophage RNA was harvested 4-6 hours post infection, as indicated, and isolated with the RNeasy kit (Qiagen) according to the manufacturer's protocol. RNA samples were treated with RQ1 DNase (Promega) prior to reverse transcription with Superscript III (Invitrogen). cDNA reactions were primed with poly dT for measurement of mature transcripts, and with random hexamers (Invitrogen) for measurement of unspliced transcripts. Quantitative PCR was performed as described [13] using the Step One Plus RT PCR System (Applied Biosystems) with Platinum Taq DNA polymerase (Invitrogen) and EvaGreen (Biotium). Transcript levels were normalized to *Rps17*. Primer sequences are listed in Table S5.

### mRNA stabilization assay

Macrophages were infected in 6-well dishes at an MOI of 1, as described above. The transcription inhibitor Actinomycin D (10 µg/mL, Sigma) was added 4 hours post infection. RNA was harvested at successive timepoints and levels of indicated transcripts were assessed by quantitative RT-PCR.

### *In vivo* experiments

Age- and sex-matched B6 or *Il23a^−/−^* mice were anesthetized with ketamine and infected intranasally with 2×10^6^ LP01 in 20 µL PBS essentially as described [13], or were treated with ExoA or Pam3CSK4 in 25 µL PBS. Bronchoalveolar lavage was performed 24 hours post infection by introducing 800 µL PBS into the trachea with a catheter (BD Angiocath 18 g, 1.3×48 mm). Lavage fluid was analyzed by ELISA. Total host cells in the lavage were counted on a hemocytometer. For RT-PCR experiments, all lavage samples receiving identical treatments were pooled, and RNA was isolated from the pooled cells using the RNeasy Kit as described above. FACS analysis of lavage samples labeled with anti-GR-1-PeCy7 and anti-Ly6G-PE (eBioscience) indicated that most cells in lavage were neutrophils. CFU were enumerated by hypotonic lysis of host cells in the lavage followed by plating on CBYE plates.

### Western blots

Macrophages were plated in 6 well dishes at a density of 2×10^6^ cells per well and infected at an MOI of 2. For whole cell extract, cells were lysed in RIPA buffer supplemented with 2 mM NaVO_3_, 1 mM PMSF, 1 mM DTT, and 1 X Complete Protease Inhibitor Cocktail (Roche). For nuclear translocation experiments, nuclear and cytosolic fractions were obtained using the NE-PER kit (Pierce) according to the manufacturer's protocol. Protein levels were normalized using the micro-BCA kit (Pierce) and then separated on 10% NuPAGE bis-tris gels (Invitrogen). Proteins were transferred to PVDF membranes and immunoblotted with antibodies to IκBα, NF-κB p65, lamin-B or β-actin (all Santa Cruz).

### ELISA

Macrophages were plated in 24 well dishes at a density of 5×10^5^ cells per well and infected at an MOI of 1. After 24 h, supernatants were collected, sterile-filtered, and analyzed by ELISA using paired GM-CSF antibodies (eBioscience). For quantification of intracellular GM-CSF, ELISAs were performed using cytoplasmic extract of macrophages infected for 6 h with the indicated strains. Levels of GM-CSF were normalized to total protein concentration. Recombinant GM-CSF (eBioscience) was used as a standard.

### Growth in bone marrow derived macrophages

Intracellular bacterial growth of wildtype and mutant *L. pneumophila* was evaluated in A/J macrophages as described [24].

### Growth in amoebae

*D. discoideum* was plated into 24-well plates at a density of 5×10^5^ cells per well in MB medium (modified HL-5 medium, without glucose and with 20 mM MES buffer) three hours before infection with the indicated *L. pneumophila* strains at an MOI of 0.05. The plates were spun at 1000 rpm for 5 minutes and incubated at 25°C. After two hours, wells were washed 3X with PBS to synchronize the infection. At successive time points, infected cells were lysed with 0.2% saponin and bacterial growth was determined by plating on growth medium.

### Protein synthesis assay

2×10^6^ macrophages were seeded in 6-well plates and infected with bacterial strains at an MOI of 2. After 2.5 h, the infected cells were incubated with 1 µCi ^35^S-methionine (Perkin Elmer) in RPMI-met (Invitrogen). After chase-labeling for an hour, the cells were washed 3× with PBS, lysed with 0.1% SDS and precipitated with TCA [24]. The protein precipitates were filtered onto 0.45 mm Millipore membranes and washed twice with PBS. Retained ^35^S was determined by a liquid scintillation counter.

### Cytotoxicity assay

Macrophages were plated in 96 well dishes at a density of 5×10^4^ cells per well and infected at an MOI of 1. At successive timepoints, Neutral Red (Sigma) was added to a final concentration of 1% and incubated for 1 h. Cells were then washed with PBS, photographed, and counted [14].

## Supporting Information

Figure S1 Genetic maps of the five deleted effectors. Numbers refer to the nucleotide position in the published *L. pneumophila LP01* genome (GenBank Accession #AE017354).
(0.67 MB TIF)

Figure S2 New transcription and mRNA stabilization of ETR target genes. (A) After a 6h infection in B6 macrophages, *de novo* transcription of the indicated genes was measured by quantitative RT-PCR with primers that specifically targeted the pre-spliced mRNA. (B) To assess RNA stability, the transcription inhibitor Actinomycin D (10μg/mL) was added to macrophages 4h post infection. RNA was collected at successive timepoints, and transcripts were measured by quantitative RT-PCR. Results are representative of two to three experiments (mean ± sd).
(0.56 MB TIF)

Figure S3 Cytotoxicity assay and measurement of intracellular GM-CSF in macrophages infected with Δ*flaA* or Δ*5*Δ*flaA L. pneumophila*. (A) B6 macrophages were infected at an MOI of 1. At indicated timepoints, the number of surviving cells was determined by Neutral Red assay. Bacteria lacking flagellin were used to avoid caspase-1-dependent cell death. (B) Intracellular GM-CSF levels were measured by performing ELISA on cytoplasmic extracts of macrophages infected for 6h with the indicated strains. Results are representative of two experiments (mean ± sd in A).
(0.60 MB TIF)

Figure S4 Induction of *Il23a*, *Gem*, and *Csf2* in dendritic cells occurs independently of Type IV secretion. B6 bone marrow derived dendritic cells were infected with the indicated strains at an MOI of 2. After 6h, RNA was harvested and transcripts were measured by quantitative RT-PCR. Results are representative of two experiments (mean ± sd).
(0.52 MB TIF)

Figure S5 *In vivo* induction of *Csf2* and *Gem* by translation inhibition. Quantitative RT-PCR measurement of *Csf2* and *Gem* expression in bronchoalveolar lavage cells collected from mice 24h after intranasal treatment with ExoA and/or Pam3CSK4. Results are representative of two experiments (mean ± sd).
(0.47 MB TIF)

Table S1 Genes induced or repressed twofold or more in *caspase-1^−/−^* macrophages infected with wildtype or Δ*dotA L. pneumophila*.
(0.31 MB XLS)

Table S2 Genes induced or repressed twofold or more in *caspase-1^−/−^* macrophages infected with wildtype or Δ*flaA L. pneumophila*.
(0.32 MB XLS)

Table S3 Deleted gene information and deletion primers for the Δ*5* strain.
(0.04 MB DOC)

Table S4 Genes induced or repressed twofold or more in *caspase-1^−/−^* macrophages infected with wildtype or Δ*5 L. pneumophila*.
(0.43 MB XLS)

Table S5 Quantitative RT-PCR primer sequences used in this study.
(0.04 MB DOC)
