# Supplementary figures and images for: Secreted Bacterial Effectors That Inhibit Host Protein Synthesis Are Critical for Induction of the Innate Immune Response to Virulent Legionella pneumophila
Source: PLoS Pathog. 2011 Feb 17;7(2):e1001289. doi: 10.1371/journal.ppat.1001289 (PMC3040669; doi:10.1371/journal.ppat.1001289)

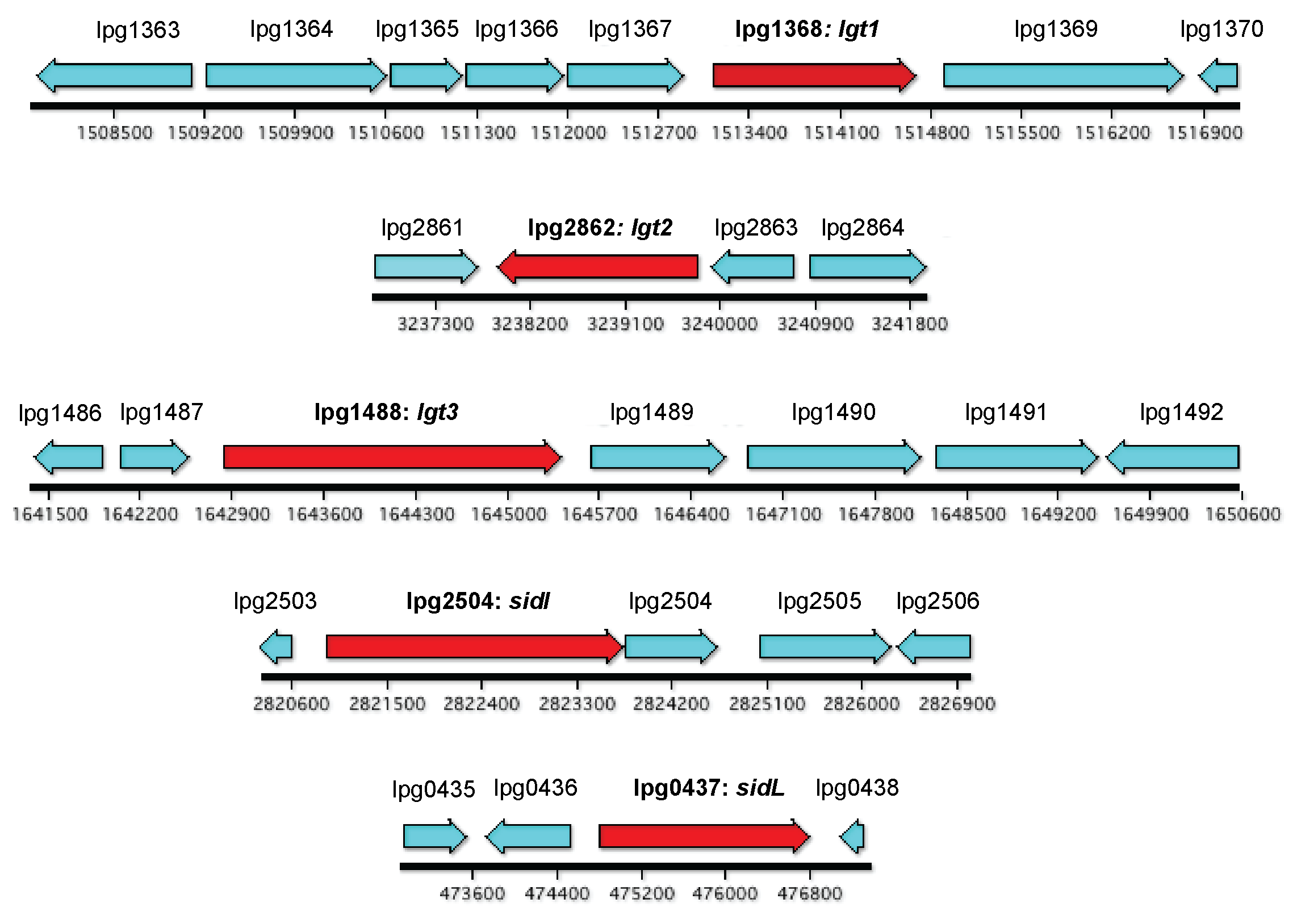

Supplement: Figure S1 — Genetic maps of the five deleted effectors. Numbers refer to the nucleotide position in the published L. pneumophila LP01 genome (GenBank Accession #AE017354). (0.67 MB TIF) [file ppat.1001289.s001.tif]

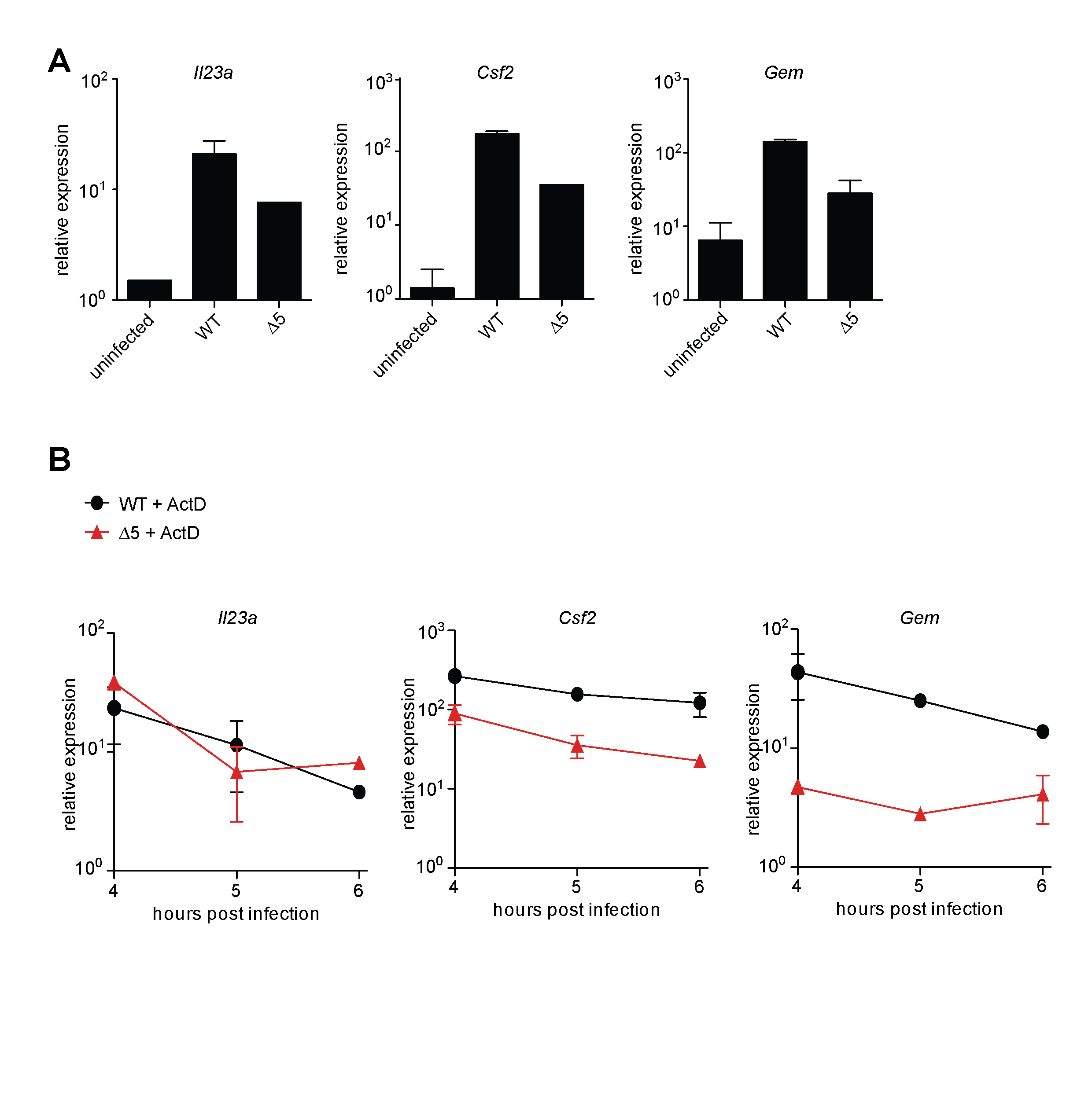

Supplement: Figure S2 — New transcription and mRNA stabilization of ETR target genes. (A) After a 6h infection in B6 macrophages, de novo transcription of the indicated genes was measured by quantitative RT-PCR with primers that specifically targeted the pre-spliced mRNA. (B) To assess RNA stability, the transcription inhibitor Actinomycin D (10μg/mL) was added to macrophages 4h post infection. RNA was collected at successive timepoints, and transcripts were measured by quantitative RT-PCR. Results are representative of two to three experiments (mean ± sd). (0.56 MB TIF) [file ppat.1001289.s002.tif]

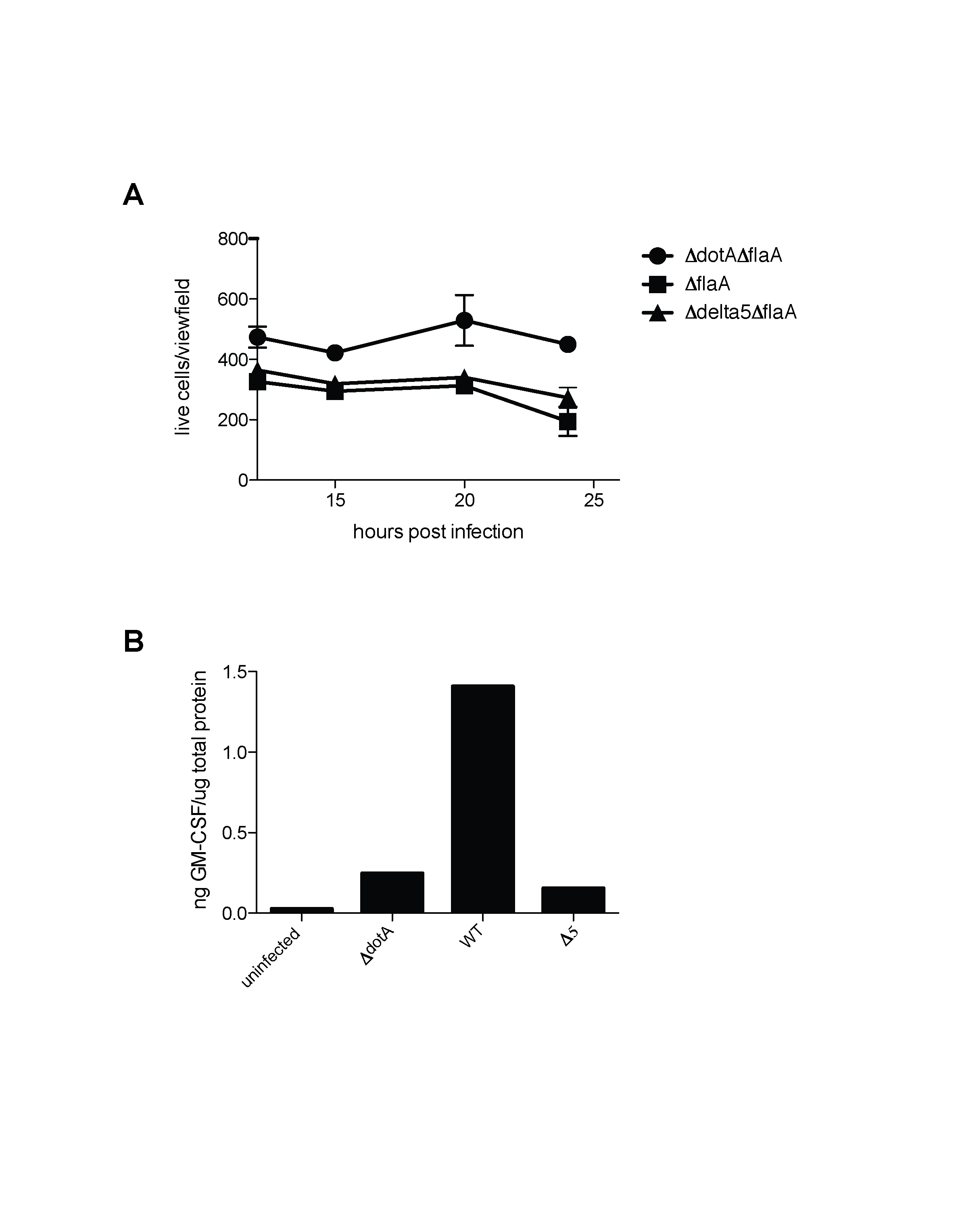

Supplement: Figure S3 — Cytotoxicity assay and measurement of intracellular GM-CSF in macrophages infected with ΔflaA or Δ5ΔflaA L. pneumophila. (A) B6 macrophages were infected at an MOI of 1. At indicated timepoints, the number of surviving cells was determined by Neutral Red assay. Bacteria lacking flagellin were used to avoid caspase-1-dependent cell death. (B) Intracellular GM-CSF levels were measured by performing ELISA on cytoplasmic extracts of macrophages infected for 6h with the indicated strains. Results are representative of two experiments (mean ± sd in A). (0.60 MB TIF) [file ppat.1001289.s003.tif]

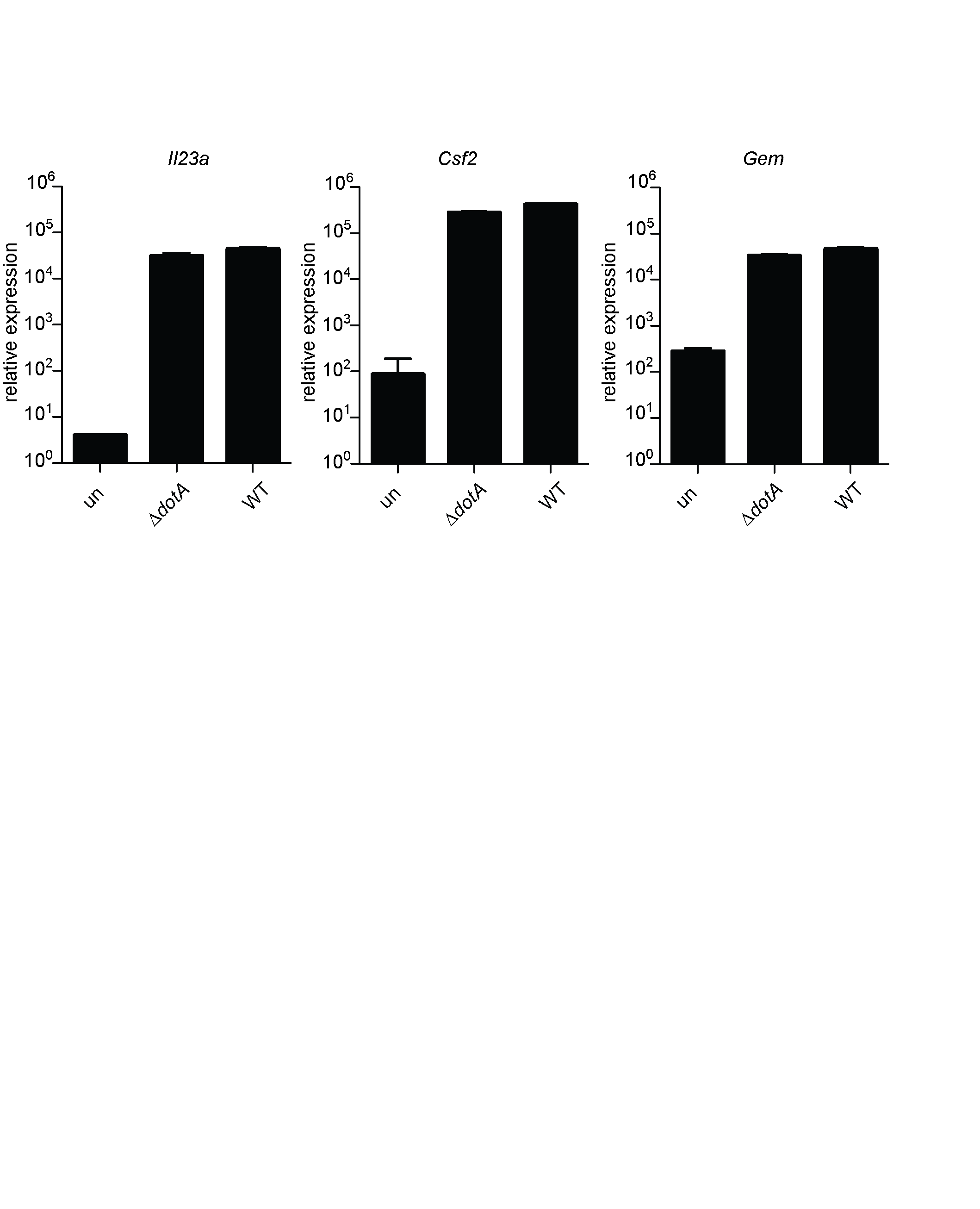

Supplement: Figure S4 — Induction of Il23a, Gem, and Csf2 in dendritic cells occurs independently of Type IV secretion. B6 bone marrow derived dendritic cells were infected with the indicated strains at an MOI of 2. After 6h, RNA was harvested and transcripts were measured by quantitative RT-PCR. Results are representative of two experiments (mean ± sd). (0.52 MB TIF) [file ppat.1001289.s004.tif]

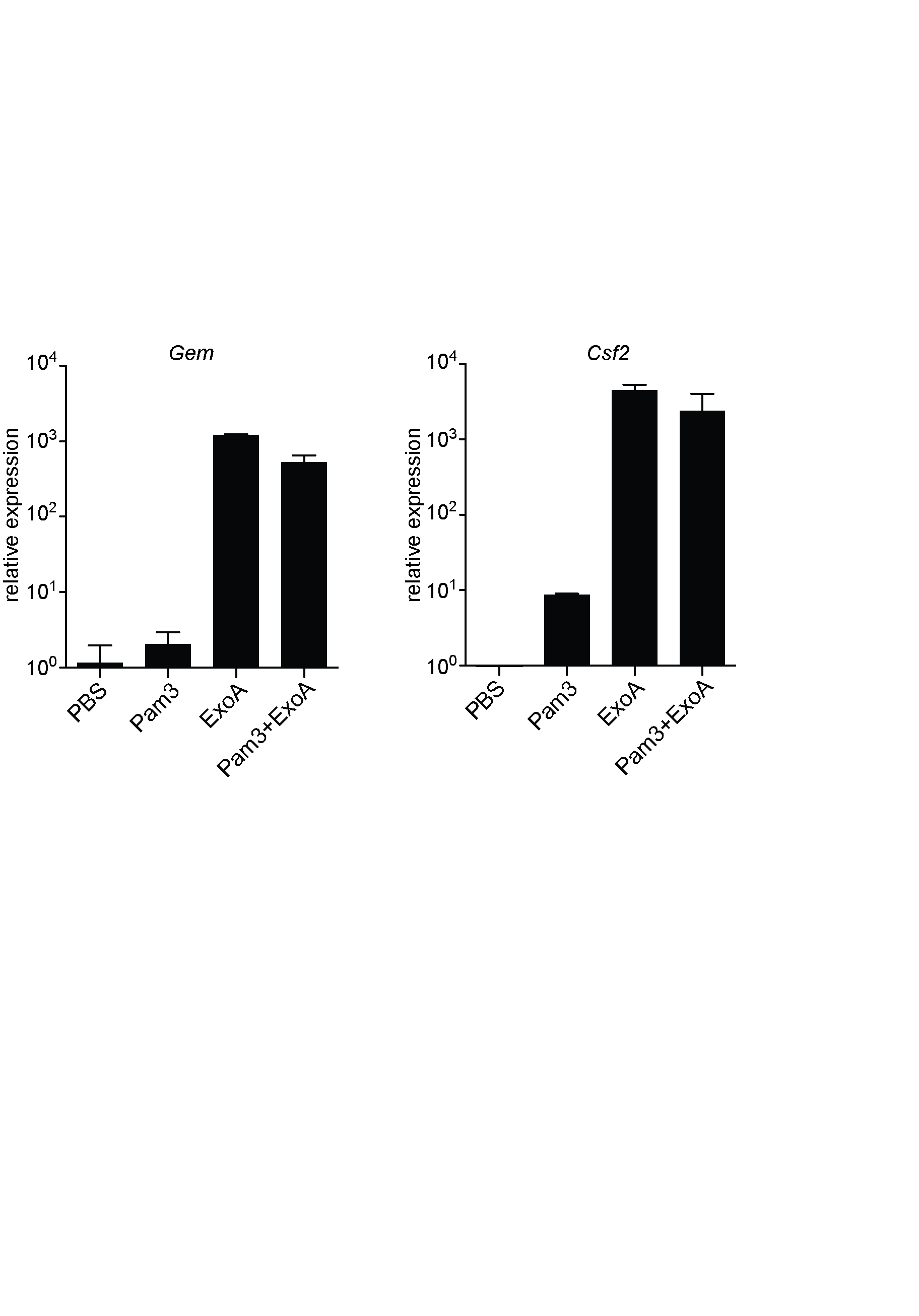

Supplement: Figure S5 — In vivo induction of Csf2 and Gem by translation inhibition. Quantitative RT-PCR measurement of Csf2 and Gem expression in bronchoalveolar lavage cells collected from mice 24h after intranasal treatment with ExoA and/or Pam3CSK4. Results are representative of two experiments (mean ± sd). (0.47 MB TIF) [file ppat.1001289.s005.tif]
