# Supplementary material for: Secreted Bacterial Effectors That Inhibit Host Protein Synthesis Are Critical for Induction of the Innate Immune Response to Virulent Legionella pneumophila
Source: PLoS Pathog. 2011 Feb 17;7(2):e1001289. doi: 10.1371/journal.ppat.1001289 (PMC3040669; doi:10.1371/journal.ppat.1001289)
Supplement: Table S3 — Deleted gene information and deletion primers for the Δ5 strain. (0.04 MB DOC) [file ppat.1001289.s008.doc]

**Supplementary Table 3.**

| **Effector** | **Gene Annotation** | **Deletion Primers** | **Reference** |
| --- | --- | --- | --- |
| Lgt1 | Lpg1368 | Upstream F: CCTGGTCGACAATACAGCAGTTTAATCC, Upstream R: CCCGGATCCTAGAGAGTTGTTGATCCC, Downstream F: CCCGGATCCGAGGGTTTGAGTTGGTTG,  Downstream R: CCAGAGCTCTGCTTATAGTCTTCTTCG | 25 |
| Lgt2 | Lpg2862 | Upstream F: AAAGAGCTCACTCGAATAGTACGGGGG  Upstream R: AAAGGATCCTTACATCCACCCTCAGTC  Downstream F: AAAGGATCCCTGCGAACGGATGGCTTG  Downstream R: AAAGTCGACCGTAGCAATGCCTAGCAG | 23 |
| Lgt3 | Lpg1488 | Upstream F: AAAGTCGACTCTCTTTGTTTTTCAAGAG  Upstream R: AACGGATCCTGTCGATTTGATATTCTC  Downstream F: AAAGGATCCCAATCTCAAAATCTTGAAG  Downstream R: AAAGAGCTCGTGATGTAGGAGAACTTG | 23 |
| SidI | Lpg2504 | See reference | 24 |
| SidL | Lpg0437 | Upstream F: GGGGTCGACGAGAGTTATATATGAGAG  Upstream R: CTCGGATCCAGATCTTTTCTCAGTTTC  Downstream F: CCAGGATCCACGACCATACGCCAAAAG  Downstream R: GCGGAGCTCTGATGACCCAAGTCTATG | this study |
