# Supplementary material for: Secreted Bacterial Effectors That Inhibit Host Protein Synthesis Are Critical for Induction of the Innate Immune Response to Virulent Legionella pneumophila
Source: PLoS Pathog. 2011 Feb 17;7(2):e1001289. doi: 10.1371/journal.ppat.1001289 (PMC3040669; doi:10.1371/journal.ppat.1001289)
Supplement: Table S5 — Quantitative RT-PCR primer sequences used in this study. (0.04 MB DOC) [file ppat.1001289.s010.doc]

**Supplementary Table 5. Quantitative RT-PCR primers used in this study.**

| **Gene** | **Sense Primer** | **Antisense Primer** |
| --- | --- | --- |
| *Rps17* | CGCCATTATCCCCAGCAAG | TGTCGGGATCCACCTCAATG |
| *IL23a* | CTAAAAATAATGTGCCCCGTATCC | GCTCCCCTTTGAAGATGTCAGAG |
| *Gem* | ACTGTGAGGTCTTGGGAGAAGA | AGAGTAGACGATCAGATAGGCAT |
| *Csf2* | GAAGAGGTAGAAGTCGTCTCTA | TATGTCTGGTAGTAGCTGGC |
| *Ifnb* | AAACTCATGAGCAGTCTGCA | AGGAGATCTTCAGTTTCGGAGG |
| *Il1b* | GACCTGTTCTTTGAAGTTGACGG | TGTCGTTGCTTGGTTCTCCTTG |
| *Nfkbia* | TGACTTTGGGTGCTGATGTC | AAGCTGGTAGGGGGAGTAGC |
| prespliced *IL23a* | CTAAAAATAATGTGCCCCGTATCC | AGCTAAATGGCCATGGACGCT |
| prespliced *Gem* | CAGTTTTCTTGTTTTCTCTCACTTG | AGAGTAGACGATCAGATAGGCAT |
| prespliced *Csf2* | GAAGAGGTAGAAGTCGTCTCTA | ACTGGCAGGGAAGAGTGTA |
